# Editorial: Mycobacteria-Host Interactions: Genetics, Immunity, Pathology

**DOI:** 10.3389/fcimb.2020.611216

**Published:** 2020-10-30

**Authors:** Alexander S. Apt, Igor Kramnik, David Neil McMurray

**Affiliations:** ^1^ Central Tuberculosis Research Institute, Moscow, Russia; ^2^ Boston University, Boston, MA, United States; ^3^ Texas A & M Health Science Center, Bryan, TX, United States

**Keywords:** mycobacteria, pathogenesis, immunity, genetic, vaccine

This research topic consists of 13 manuscripts that focus on some aspect of the interaction between mycobacteria and their hosts. Many report novel experimental data, while others are minireviews of the pertinent literature. A few of the manuscripts reflect the unique perspectives of the authors on issues in tuberculosis (TB) of some controversy.

For purposes of this editorial, we have organized the manuscripts into discrete sub-topics with the indicated number of papers in each: TB-comorbidities [diabetes (2); TB and HIV (2)]; aspects of TB pathogenesis [dormancy (2) and dissemination (2)]; innate (2) and adaptive (2) immunity; and a provocative paper on the application of personalized or precision medicine in TB (1).

Segura-Cerda et al. have reviewed the literature that establishes Type 2 diabetes (T2D) as an important co-morbidity that increases the risk 2–4 fold for the development of pulmonary TB. In this manuscript, the authors focus on the putative mechanisms that might explain this synergistic relationship. They highlight dyslipidemia and hyperglycemia as two of the conditions associated with T2D that exacerbate TB. Several hormonal changes (e.g., decreases in leptin, ghrelin, and reactive oxygen species) and vitamin D deficiency are also discussed as they relate to increased susceptibility to TB. A related paper (Kumar et al.) reports the significant associations that they observed between plasma eicosanoid levels, lung pathology and bacterial burden in human patients with TB–T2D co-morbidity and speculate about potential mechanisms.

HIV infection is a risk factor for relapse following failed chemotherapy in treated TB patients, but the mechanisms involved are not understood. Huante et al. have adapted a humanized mouse model of TB–HIV co-infection to study the effect of HIV on relapse in mice with post-chemotherapy pauci-bacillary infection. The authors used the so-called “Cornell” approach to drive infection levels down with rifampin and isoniazid, and then infected the mice with HIV. Relapsing co-infected mice exhibited increase bacterial and viral burdens in the lungs. This novel model has great potential to elucidate the synergistic effects of these two pathogens on the host. In a second paper related to TB–HIV co-infection, Naqvi and Endsley review the roles of C-type lectin receptor (CLR) recognition. CLRs such as the mannose receptor, Mincle, Dectin 1 & 2, DC-SIGN, and others may play important roles in protective immunity and immune evasion in TB/HIV and their genetic polymorphisms in populations may influence disease susceptibility in co-infected individuals.

There is perhaps no aspect of TB pathogenesis more intriguing or controversial than latency or dormancy. Two papers in this Research Topic address that issue. Trutneva et al. created dormant *Mycobacterium tuberculosis* H37Rv using established culture procedures and studied the protein profiles of the non-replicating bacilli over a 13-month period. The proteome of the dormant bacilli differed markedly from that of metabolically active cells but, despite the substantially diminished size of the dormant cells, they contained numerous intact proteins. The authors speculate that the presence of chaperones, DNA-stabilizing proteins, and enzymes involved in protection from oxidative stress were responsible for the preservation of many proteins in dormant *M. tuberculosis*. In the second paper, Salina et al. identified an NO-inducible small mycobacterial RNA (MTS1338) that apparently regulates a shift in the transcriptome profile consistent with adaptation to the environment within the macrophage. The authors speculate that MTS1338 may be involved in the transition to the non-replicating, dormant state within host cells.

Although pulmonary disease is the most common clinical presentation of TB, there are several extra-pulmonary manifestations that present both diagnostic and therapeutic challenges. Moule and Cirillo have reviewed the literature on disseminated TB [e.g., lymphadenitis, spinal infection (Pott’s Disease), pleurisy, and central nervous system (CNS) disease] and have focused on the mechanisms by which mycobacteria may escape from the lung. They examine the potential roles of lung epithelial cells, dendritic cells, alveolar and infiltrating macrophages in the escape of bacilli from the lung and suggest that extra-pulmonary spread may be a common consequence of all TB lung infections. A second paper (Kumar et al.) focuses on CNS TB using a well-established rabbit model of meningitis in which different strains of *M. tuberculosis* were injected intra-cisternally. The authors report that blocking pro-inflammatory cytokines (e.g., TNFα, IL-6, IL-1β) improves the clinical outcome and suggest that phosphodiesterase-4 inhibitors may have therapeutic potential for treatment of humans with TB meningitis.

Four of the manuscripts in this Research Topic discuss the mechanisms by which innate host responses and adaptive immunity contribute to a successful host response to mycobacterial infection. Two of the papers have examined the role of Th-17 cells that produce IL-17A, a cytokine which plays a protective role early in infection but drives tissue destruction later in the course of disease when it becomes unregulated. Leisching reviews the importance of PI3-Kinase pathways in driving a Th-17 response and that the p110δ and p110γ isoforms have opposite effects on Th-17 cell populations. The author speculates that inhalable PI3-Kinase inhibitors might have therapeutic benefit in TB by dampening the unregulated IL-17A response. Huang et al. report that Interleukin-2-inducible T-cell kinase (ITK) is involved in early protection against pulmonary TB in mice. ITK-deficient mice developed increased bacterial burdens and pathology in the lungs accompanied by a defect in the development of IL-17A-producing γδT cells. They also demonstrated that pulmonary granulomas from active TB patients contained increased levels of ITK mRNA compared to normal lung tissue using laser capture microdissection. The authors suggest that enhancing ITK activity may be a viable host-directed therapeutic approach.

Kang et al. utilized a novel ethyl cinnamate method for clarifying mouse lung tissues to examine the localization of different types of innate immune cells (e.g., neutrophils, alveolar and infiltrating macrophages, etc.) within pulmonary granulomas. The mice were infected with *M. tuberculosis* by the aerosol route and the lungs were removed after 4 weeks. The clarified tissues were imaged using light sheet fluorescence microscopy and 3D images of the infected lungs were created.

Le Moigne et al. used both *in vitro* and *in vivo* approaches to examine the involvement of a TLR-2 activating factor in the pathogenesis of *Mycobacterium abscessus*, an important pulmonary pathogen in cystic fibrosis (CF) patients. Antibodies against the factor were found in the sera of CF patients. However, vaccination with the TLR-2 activating factor (a mixture of several lipoproteins) exhibited no protection against aerosol infection of mice with *M. abscessus*, but provided modest protection when mice were challenged intravenously.

The final paper in this Research Topic is a thought-provoking minireview/perspective written by Azad et al. The authors have reviewed the genetic basis for considerable human-to-human variation in cellular, inflammatory and immune responses to TB. They advocate for a “personalized” or “precision” medicine approach to compensate for those differences but admit that this approach is fraught with challenges. These include the lack of sufficient genetic data in many ethnic groups and the difficulty in delivering individualized therapies in high-burden, low-resource settings.

We believe that the manuscripts in this Research Topic reflect novel approaches to filling some of the most vexing gaps in our understanding of the interactions between mycobacteria and their hosts. Several of the papers have clear translational significance which bodes well for the application of this knowledge to improved TB prevention and control going forward.

## Author Contributions

All three authors contributed equally to this editorial. All authors contributed to the article and approved the submitted version.

## Conflict of Interest

The authors declare that the research was conducted in the absence of any commercial or financial relationships that could be construed as a potential conflict of interest.

